# Development, Verification and Assessment of a Laser Profilometer and Analysis Algorithm for Microtexture Assessment of Runway Surfaces

**DOI:** 10.3390/s24237661

**Published:** 2024-11-29

**Authors:** Gadel Baimukhametov, Greg White

**Affiliations:** School of Science, Technology and Engineering, University of Sunshine Coast, Sippy Downs, QLD 4556, Australia; gadelbaimukhametov@gmail.com

**Keywords:** laser profilometer, texture, microtexture, macrotexture, filtration algorithm, British pendulum tester, friction

## Abstract

Runway surface friction is critically important to safe aircraft operations and mostly depends on the surface texture, which provides grip in the presence of contamination and directly affects the friction coefficient in general. Microtexture assessment is the most challenging part of texture assessment since there is no standardised pavement microtexture control method in runway maintenance and management practice. The purpose of this study was to develop a simple laser profilometer and analysis model and subsequent validation for use in runway friction surveys. To that end, a simple laser profilometer was developed, and a profile picture analysis and macrotexture filtration method were designed. Test results were compared to the stylus-based roughness tester and the British Pendulum Tester. The proposed profile picture analysis and profile smothering and filtration methodology, based on linear approximation, is simpler and more effective for the case of macrotexture filtration for the friction survey. The laser profilometer model results were highly correlated with the stylus-based roughness tester results (R^2^ = 0.99). The average roughness of the microtexture profile, after smothering and macrotexture filtration, also showed good correlation with the British Pendulum results (R^2^ = 0.78). The results from this study confirm the possibility of texture assessment for routine runway friction surveys using a simple and economical laser profilometer, which is not routinely available in current airport surface friction management.

## 1. Introduction

**Figure 1:**
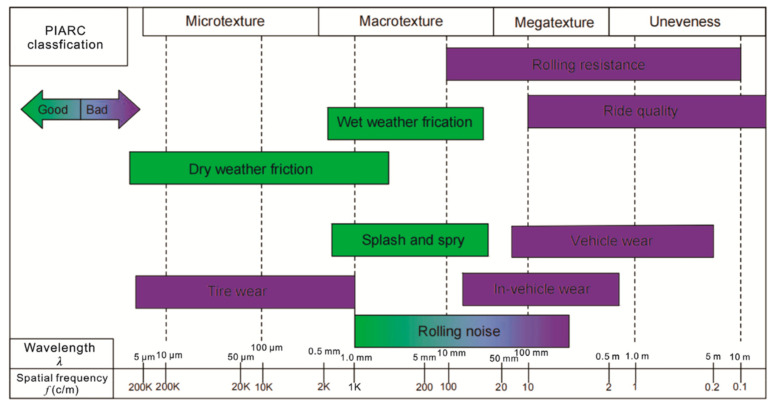
PIARC classification for pavement surface characteristics according to wavelength [2].

Surface texture is a set of surface irregularities characterised by the size, depth and other shape parameters. It is usually divided into different classes, depending on the size of irregularities. This is important for the analysis of texture because all of the classes affect the rolling and sliding performance of the tire differently. As shown in Figure 1, the Permanent International Association of Road Congers (PIARC) defines four classes of texture: microtexture, macrotexture, megatexture and unevenness [1]. For practical purposes, only micro- and macrotextures are important for runway surface friction management due to the tire-pavement contact area being relatively small compared to megatexture and unevenness.

The macrotexture of a runway surface mostly depends on the type of surface, being a mixture type, and maximum aggregate size. Open-graded asphalt mixes, stone mastic asphalt, and porous asphalt usually have a relatively high texture [3,4]. In contrast, dense-graded asphalt and cement concrete have lower macrotexture, and in most cases, special treatments are required to increase the actual or effective macrotexture [5,6]. Importantly, even when adequate macrotexture is provided at the time of surface constructure, it can be reduced due to contamination and wear of the pavement through the life of the surface [7]. Consequently, ongoing monitoring and measurement are required.

In contrast, the microtexture mostly depends on the parameters of the stone aggregate. Stone aggregate roughness can be different due to the size of the aggregate, its shape and its hardness. Crushed stone, for example, usually has a larger microtexture than rounded natural gravels [8]. The roughness of a stone can also change due to wear and erosion, a phenomenon known as aggregate polishing, with higher initial microtexture usually associated with a higher rate of microtexture wear during service [9]. Surface microtexture can also be reduced due to contamination and the “masking” effect when binder, rubber, clay, dust, or other contaminants fill the microtexture cavities and pores, reducing the microtexture [10,11]. In practice, it is common for microtexture to be revealed as the binder is worn off the exposed aggregate in a new surface [12], but then to be masked over time by rubber contamination from landing and taxiing aircraft tires [13].

Due to the evolution of both microtexture and macrotexture over the life of a runway surface, texture parameters require regular monitoring because of their impact on aircraft safety, surface durability and the associated environmental effects [14]. Although macrotexture measurements are simple and well established, with a number of recommended techniques available [2], microtexture measurements are not common and require improved standards and monitoring systems. For example, the International Civil Aviation Organisation (ICAO) does not currently recommend any microtexture measurement techniques. In their international regulation known as Annex 14 [15], it is recommended to provide a good microtexture. However, their guidance material, known as PANS Aerodromes [16], recommends only visual and tactile assessment of microtexture, which can only be used for the detection of contamination and defects.

**Figure 2:**
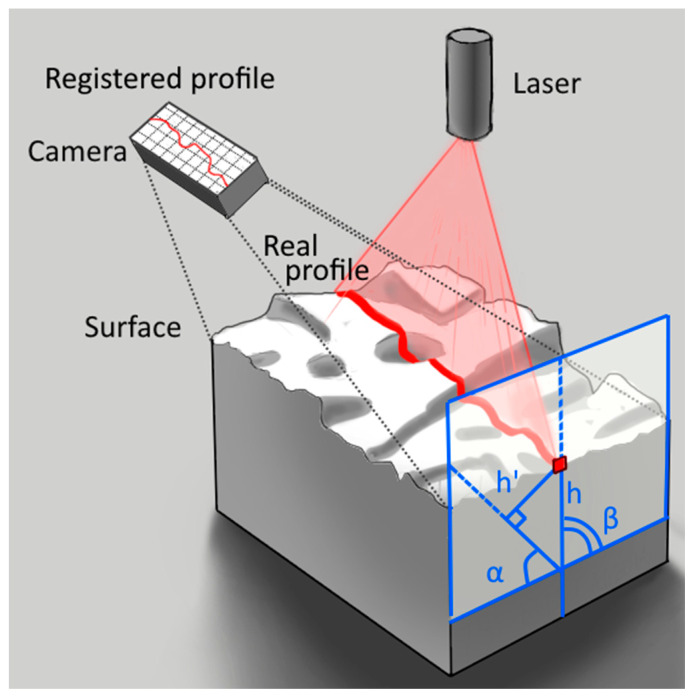
Laser profilometer.

In contrast to airport pavement practice, researchers have used different methods of microtexture assessment, which can be divided into contact and non-contact methods. Contact methods include the mechanical stylus test [17] and different wear tests [18]. Non-contact methods are more common and include laser profilometry [19], image texture analysis [20], stereoscopy [21], computer tomography scanning [22], 3D scanning [23] and simpler microscopy assessment methods, such as the straightedge shadow method [9,24]. The most precise and simple method is laser profilometry, which is based on the geometrical measurement of a projection of the laser beam on a surface [25]. Laser profilometry requires a profilometer, the main components of which are the laser and a camera (Figure 2), and data filtration and analyses software.

The relative height of the profile point on the surface and on the registered or measured profile shown in Figure 2 are related by Equation (1).

(1) $$h=\frac{h^{\prime }\cdot \sin\beta }{\sin\left(180-\alpha -\beta \right)}$$

where *h* is a real height of the point on a profile, *h*′ is a height of the point on a registered profile, *α* is an angle between camera and surface and *β* is an angle between laser and surface.

Surface texture parameters can be separated into two-dimensional amplitude and shape-related parameters, three-dimensional amplitude and shape-related parameters, spectral characteristics of a surface wave and fractal and multifractal characteristics [2]. Amplitude and shape-related parameters are widely used during the texture assessment. However, the other parameters have limited use in regards to the texture analysis of pavement surfaces. In one study [26], macrotexture, obtained by a circular track meter, was characterised using wavelet analysis, which allowed the analysis of the influence of different aggregate sizes on surface texture. In a similar study [27], asphalt surface friction was characterised as a set of fractal parameters of texture. Fractal mathematics was also used for the texture analysis by Kikkalis et al. [28]. Besides that, fractal surface parameters can be effectively used for the surface topography analysis in material studies [29]. In this study, however, the surface texture for simplicity was characterised using the average roughness of a profile.

Various researchers have studied the influence of texture on available surface friction [30,31,32,33]. However, only a few of them have included microtexture measurements for the friction prediction (Table 1).

Some of the abovementioned studies use theoretical models for friction calculations [36,37,39]. These studies are generally based on a hysteresis friction model [35]. This model also allows for the inclusion of contamination, such as water film thickness, using the same textural parameters, assuming that contamination acts as a filler for the texture and reduced the height of the asperities. Those models also showed good correlation with CFME data. A Persson model can also be combined with numerical analysis to calculate the stresses in the contact area and increase the reliability of the model [45]. However, practical use of those models is difficult since they require complex numerical analysis and a detailed survey of the surface texture, and it can be seen that the study based on the data obtained from field evaluation of the pavement parameters shows a lower accuracy than required [39].

In some of the more recent studies [42,43], artificial neural networks were used for friction prediction. An artificial neural network is a good tool for friction analysis, and it shows a high correlation between predicted and measured values for some pavements. However, one of the drawbacks of neural network models is that they work as a ‘black box’, since the calculation process is not presented for analysis. That creates a risk because the result is based mostly on the training process and some properties of materials used for training and not on fundamental dependencies [46].

Most of the abovementioned studies use 3D scanning for the microtexture measurements. That technique, however, requires expensive testing equipment and cannot be used widely in the field. Only three studies [36,38,44] used laser profilometry for microtexture assessment, with the equipment being different in all three cases. The parameters used in these studies were also different, which makes the comparison of the results difficult. Two of the studies [38,44] used linear models for the pavement friction prediction, the appropriateness of which also needs to be validated.

In general, recent research analysis shows that texture assessment can reliably predict surface friction, including the effect of contaminants on aircraft skid resistance. However, texture assessment methods are not fully developed within current international practice, and there is no friction model commonly used due to a lack of widely available microtexture assessment techniques of real runway pavement surfaces.

The aim of this study was to develop a simple and economical laser profilometer and to validate the potential to use that laser profilometer for surface texture characterisation and ultimately towards a unified microtexture assessment methodology, including microtexture profile analysis. For this purpose, the laser profilometry equipment was produced and verified against a standard roughness measurement device on plane surfaces before being assessed on real pavement surfaces against spot friction, measured by a British Pendulum machine.

## 2. Materials and Methods

### 2.1. Verification Surfaces

For the verification of the profilometer, a set of surfaces with minimal macrotexture was used. Those surfaces include sandpaper with P60, P80, P120, P180, and P240 grit, polished metal plates, and a mill file. A total of 8 samples were tested using a laser profilometer and roughness tester.

### 2.2. Validation Surfaces

**Figure 3:**
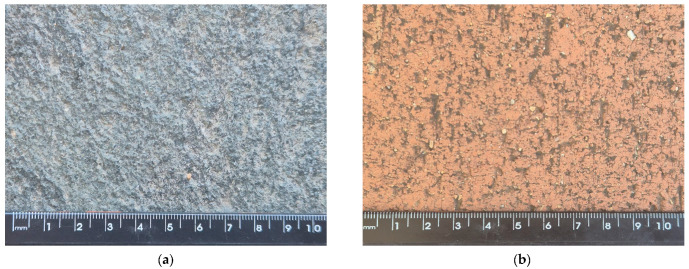

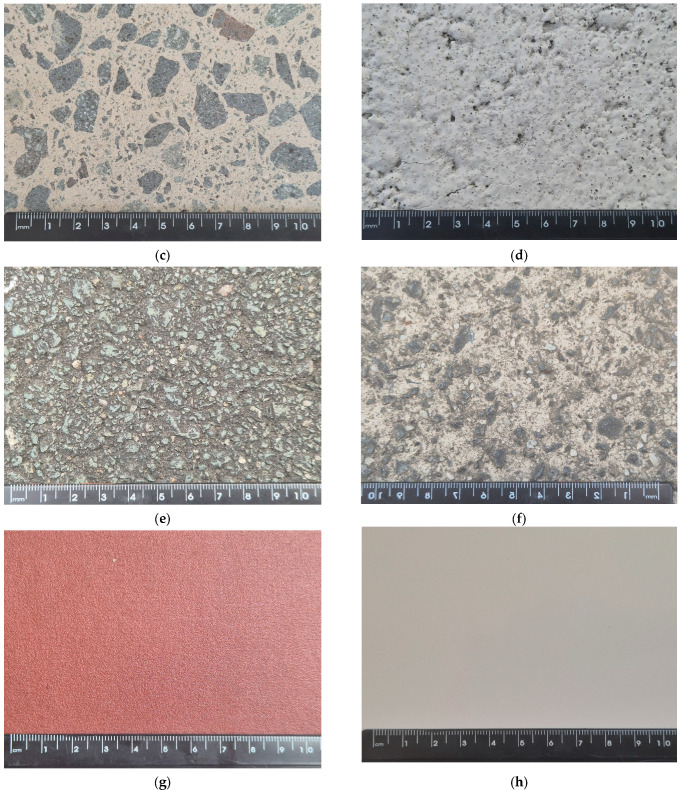
Examples of tested surfaces: (**a**) natural paving stone, (**b**) ceramic paving stone, (**c**) polished concrete, (**d**) road marking, (**e**) asphalt, (**f**) rough concrete, (**g**) sandpapers and (**h**) whiteboard.

For the microtexture and friction assessment, various surfaces were considered, including polished concrete (Figure 3c), rough concrete (Figure 3f), older asphalt (Figure 3e), road marking paint (Figure 3d), paving stones (Figure 3a,b), different grades of sandpaper (Figure 3g) and a smooth whiteboard (Figure 3h). The sandpaper and whiteboard were included to increase the range of the texture measured since all of the other surfaces had a generally similar microtexture. The sandpaper was secured to a heavy concrete slab using cyanoacrylate glue, to keep it flat and to avoid measurement errors. All of the tested surfaces had minimal macrotexture to avoid errors during the friction measurement. For this study, 17 total measurements were performed on surfaces with different microtexture values.

### 2.3. Friction Measurements

**Figure 4:**
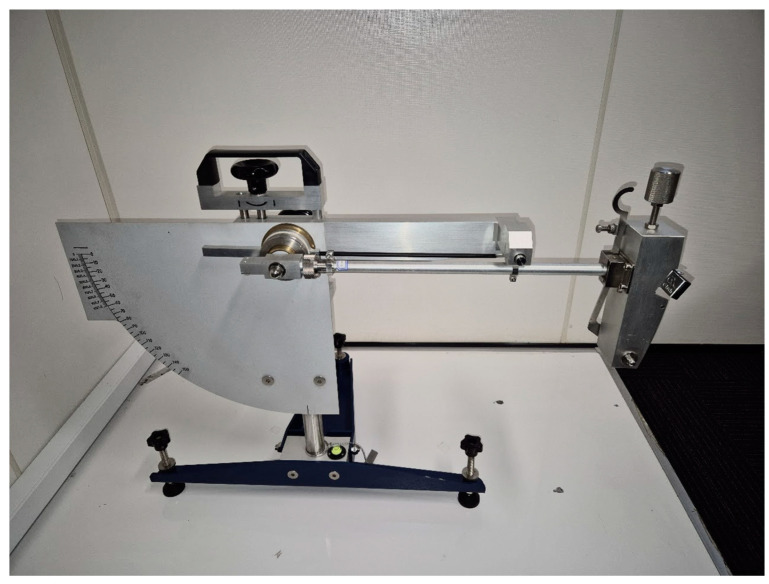
British Pendulum machine.

The British Pendulum machine was used for the friction measurements (Figure 4). This method is widely used for the friction measurement of different surfaces. This method is based on the measurement of the pendulum energy after sliding on a wet surface with the rubber slider on the end of the pendulum. The British Pendulum Number (BPN) is the unitless value shown on the plate behind the pendulum, reflecting the angle of the pendulum swing after sliding. For this study, measurements were performed on a wet surface. The reliability of the British Pendulum test has been shown to decrease with an increase in macrotexture [47]. Consequently, it is not recommended to use the British Pendulum on surfaces with a high macrotexture, such as sprayed seals and stone mastic asphalt.

### 2.4. Surface Texture Measurement

**Figure 5:**
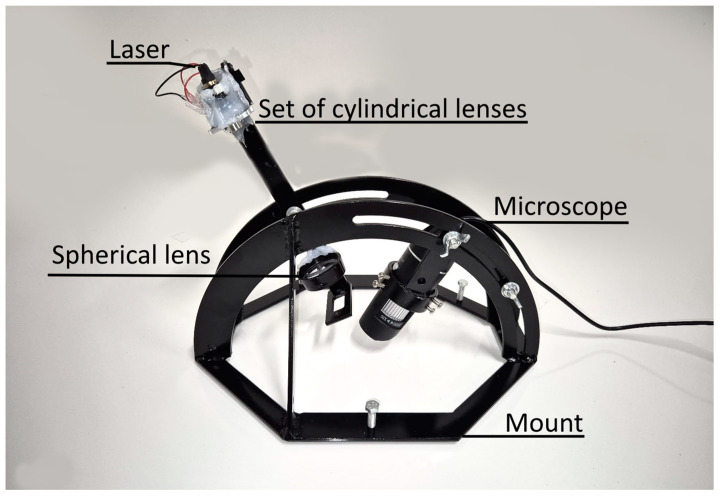
Laser profilometer for microtexture assessment.

For the surface texture measurements, a simple laser profilometer was designed (Figure 5). Although laser profilometry is a well-known tool and there are commercial laser profilometers available, this study required a new model that was suitable for pavement surface measurement. The profilometer was also designed to be economical and small in size, allowing it to be used by many airports around the world. The profilometer mount allows the angles between the laser and the camera and pavement surface to be adjusted. The main advantages of the model, compared to commercial equipment, are the component price, which is equal to 36 AUD in total, excluding the price of the mount, which was made from the 3 mm sheet metal. There are no comparable commercially available products designed for the microtexture assessment of pavement surfaces, with similar equipment costing more than 1000 AUD [48].

**Figure 6:**
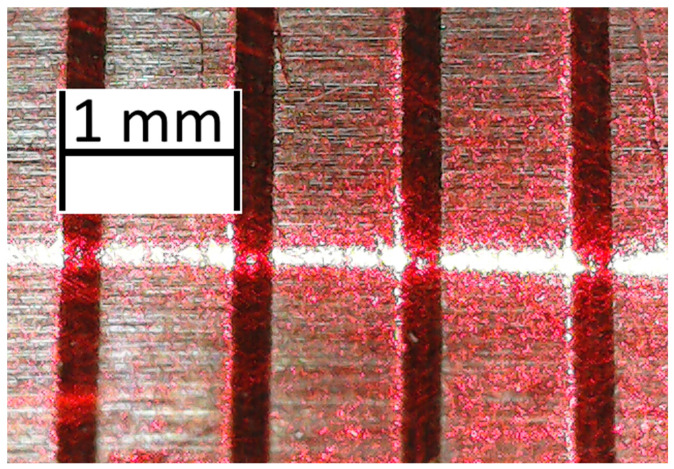
Horizontal resolution of the laser profilometer.

The horizontal resolution of the laser profilometer is equal to 6.09 µm. Vertical resolution can vary by changing the angle between the camera and the laser (1). The thickness of a laser beam is approximately 100 µm, but the real vertical resolution is finer due to the nature of the processing algorithm, as shown in the example in Figure 6.

**Figure 7:**
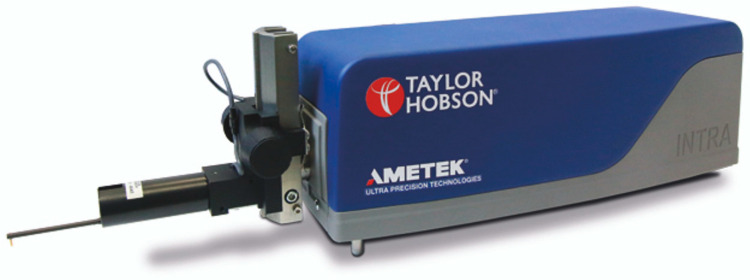
Intra Touch roughness tester.

Verification of the laser profilometer was performed using the Intra Touch roughness tester (Figure 7) with 4 nm vertical and 0.5 µm horizontal resolution. This roughness tester is based on the stylus test and allows precise texture measurement. The vertical range of that tester, however, is limited. Consequently, it is only possible to use it to measure the microtexture of plain surfaces with little or no macrotexture.

### 2.5. Data Processing

Once the surface profile is registered, it must be analysed. Consequently, a data processing algorithm was also designed. It consisted of the following four steps:

1. Profile registration.
2. Fine smothering.
3. Macrotexture filtration.
4. Texture parameters calculation.

**Figure 8:**
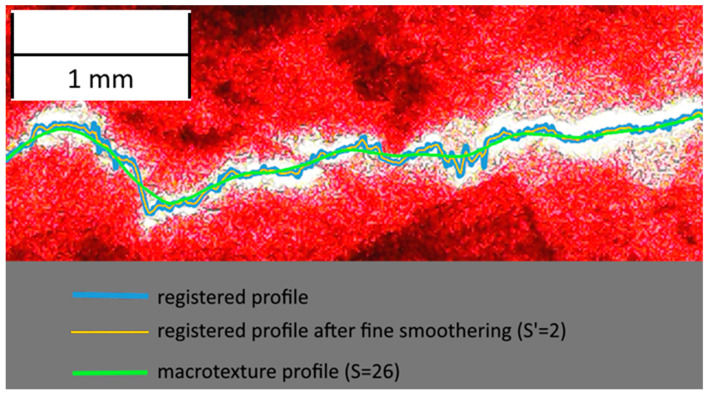
Processing of the photo of a profile.

The profile generated by the laser profilometer was first photographed with a phone-based camera. The photo was processed to obtain a texture profile. First, pixel brightness was calculated, as well as the profile line brightness threshold. After that, the points on the profile were calculated by finding the centre of brightness of each column of pixels. The registered profile was then smothered to remove any errors (Figure 8).

**Figure 9:**
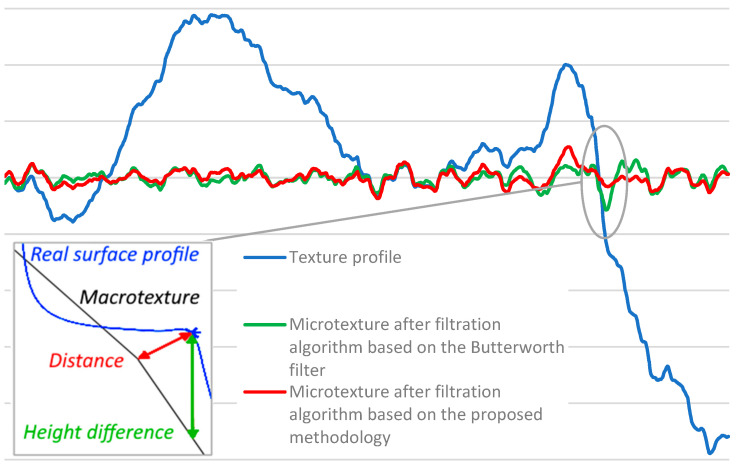
Comparison of existing and proposed algorithms [50].

The obtained profile consists of macrotexture and microtexture. Macrotexture needs to be filtrated out for the microtexture analysis. There are many algorithms for macrotexture filtration, for example, the Fourier transformation, the Butterworth filter [49] and the Gaussian smoothing filter [43]. All of these can effectively isolate the microtexture from the macrotexture. However, the main drawback of those methods is they use orthogonal filtration, which can lead to microtexture overestimation. An example of that is shown in Figure 9. In this example, two different filtration algorithms were applied to the profile obtained by Florková et al. [50]. The existing algorithm was based on the Butterworth filter, and the microtexture depth was calculated as the height difference between the macrotexture and a point on a profile. A new algorithm was also applied that uses the distance between the point on a profile and the macrotexture profile. As shown in Figure 9 in a circle, the existing algorithm overestimates the microtexture. That overestimation affects zones near the slopes of macrotexture, and in the case of surfaces with deep macrotexture, that difference can be significant. For that reason, the new algorithm is justified for use with airport pavements, where it is crucial to provide a good macrotexture.

The macrotexture profile needs to be approximated based on the profile data for the filtration. Polynomial spline approximation is smoother than a linear approximation and can be used for mean profile approximation as well. However, for this study, the linear approximation was used for a number of reasons.

**Figure 10:**
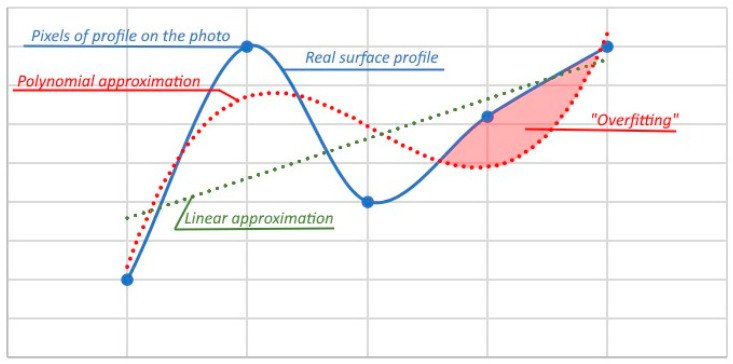
Smoothening of the profile with linear and polynomial approximations.

First, the polynomial spline is designed to “fit” the natural shape of the elastic beam, thus it can create an almost smooth profile [51]. The real profile or surface, however, is not limited by any force and can create an odd shape. Polynomial spline approximation can also lead to “overfitting”, which leads to an obvious error [52]. An example of overfitting is shown in Figure 10.

**Figure 11:**
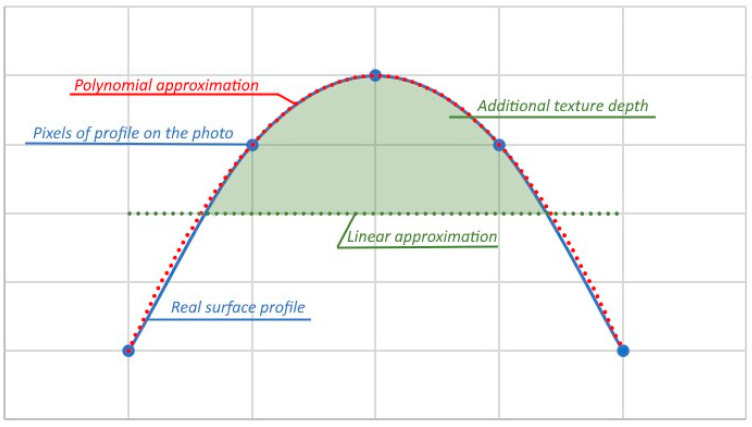
Polynomial and linear approximations for the smoothening of the profile.

Moreover, the friction problem requires an unusual solution for the profile approximation. For example, if you compare the flat surface and the surface with a curved shape, it is obvious that the grip is better on a curved surface. If we consider the flat surface to be the most slippery surface, we cannot use polynomial approximation for the smoothening of the surface. Otherwise, we will inevitably lower the overall predicted grip since we decided to characterise the grip by using the texture depth. An example of that is shown in Figure 11.

And finally, since the method in this study requires the calculation of the exact distance to the point, that problem is crucial for the acceptability of the filtration algorithm. With the polynomial approximation, it is hard to find the exact distance from any given point to the macrotexture profile. This problem can be solved using brute-force algorithms or using more advanced methods, such as the spherical clipping method [53]. All of the methods compute the approximate solution and require significant computational resources [54], especially in the case of the texture profiles, because the profile consists of thousands of points and the processing time can also become crucial. In the case of linear approximation, however, it is a simple problem with a simple solution, according to Equation (2).

(2) $$d_{n}=\frac{y_{n}-b_{n}x_{n}-a_{n}}{\sqrt{1+b_{n}^{2}}}$$

where *d_n_* is a distance between the *n*-point (*x_n_*, *y_n_*) and the line defined by Equation (3).

(3) $$y=a_{n}+b_{n}x$$

where *a_n_* and *b_n_* are coefficients of the line on a profile for each *n*-point.

In that case, the number of points for the linear approximation can be used as a filtration coefficient. The proposed algorithm finds a linear approximation based on the method of least squares for the (*n* − *S*; *n* + *S*) points for each *n*-point, where *S* is a filtration coefficient. The same algorithm is used for the fine smothering, with the number of points for linear approximation being equal to 2 × *S*′ + 1, where *S*′ is a fine smothering coefficient (Figure 8). Coefficients *a_n_* and *b_n_* (2) for each point can be found using Equation (4) and Equation (5), respectively.

(4) $$a_{n}=\frac{\sum_{i=n-S}^{n+S}y_{i}}{2S+1}-b_{n}\frac{\sum_{i=n-S}^{n+S}x_{i}}{2S+1}$$

(5) $$b_{n}=\frac{\frac{\sum_{i=n-S}^{n+S}x_{i}y_{i}}{2S+1}-\frac{\sum_{i=n-S}^{n+S}x_{i}}{2S+1}\cdot \frac{\sum_{i=n-S}^{n+S}y_{i}}{2S+1}}{\frac{\sum_{i=n-S}^{n+S}{x_{i}}^{2}}{2S+1}-{\left(\frac{\sum_{i=n-S}^{n+S}x_{i}}{2S+1}\right)}^{2}}$$

The same method can be used for the fine smothering. In the case of fine smothering, distance calculation is not important. Consequently, for simplicity, *n*-point coordinates (*y*′*_n_*, *x_n_*) of the point on a smothered profile can be calculated from Equation (6).

(6) $${y^{\prime }}_{n}=a_{n}^{\prime }+b_{n}^{\prime }x_{n}$$

where *a*′*_n_* and *b*′*_n_* can be found for each point using Equation (4) and Equation (5), respectively, and using *S*′ coefficient instead of *S*.

**Figure 12:**
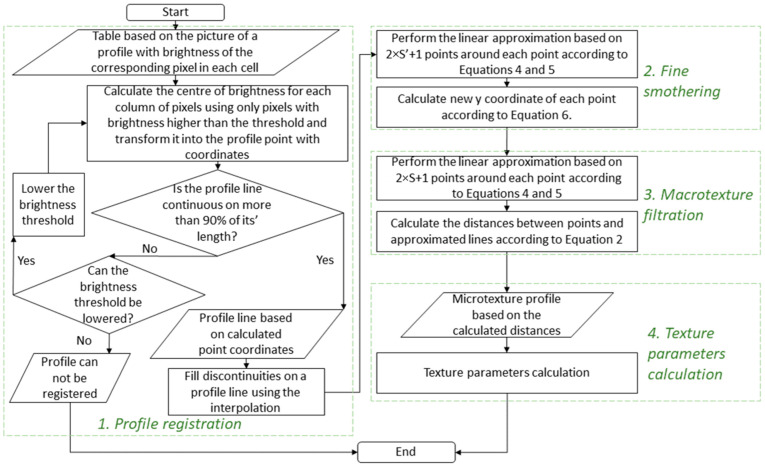
Block diagram of microtexture profile analysis algorithm.

Details of the profile registration algorithm and each further step are shown in Figure 12. This algorithm was applied using the Visual Basic application for Excel due to the simplicity of data storage and compatibility with other data analysis tools.

## 3. Results and Discussion

### 3.1. Laser Profilometer Verification

As stated above, the verification of the laser profilometer was performed using a stylus-based Intra Touch roughness tester (Figure 7). A sandpaper, smooth metal plate and a mill file were used as testing surfaces. Four sets of tests were performed with different laser and profilometer angles (Figure 2). The results are presented in Table 2. At the same time, the *S*′ coefficient was optimised to maximise the R^2^ value. The final *S*′ coefficient in all cases was 2.0. An average roughness (Ra) value was calculated in all cases without the macrotexture filtration.

**Figure 13:**
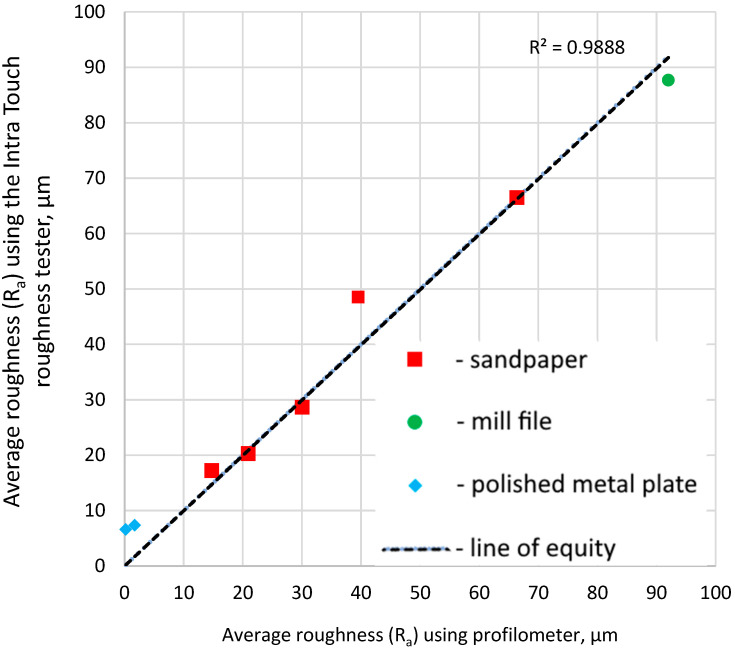
Correlation between profilometer and Intra Touch roughness for optimised laser and camera angles.

It was found that angle between camera and laser and surface affected the correlation between the roughness tester and laser profilometer results. The lower the angles, the higher the vertical resolution. However, too low an angle for the camera obstructs the vision of the asperities of macrotexture. Furthermore, too low an angle for the laser distorts the profile projection. The maximum correlation between the roughness tester and profilometer was obtained in the fourth series of tests, with both angles being set to 60°. The associated correlation is shown in Figure 13. Despite the high correlation indicating the profilometer is in fact valid, it can be seen that the resolution of the laser is lower than that of the Intra Tech roughness meter, because at average roughness (R_a_) lower than 10 μm, the profilometer tends to underestimate the Intra Tech reported roughness. Despite this, the results are proportionally consistent for the two devices. This error for finer surfaces, however, will systematically occur for such laser profilometers. This problem, however, can generally be ignored for runway friction management due to the coarseness of pavement surfaces.

### 3.2. Laser Profilometry for Surface Texture Assessment

**Figure 14:**
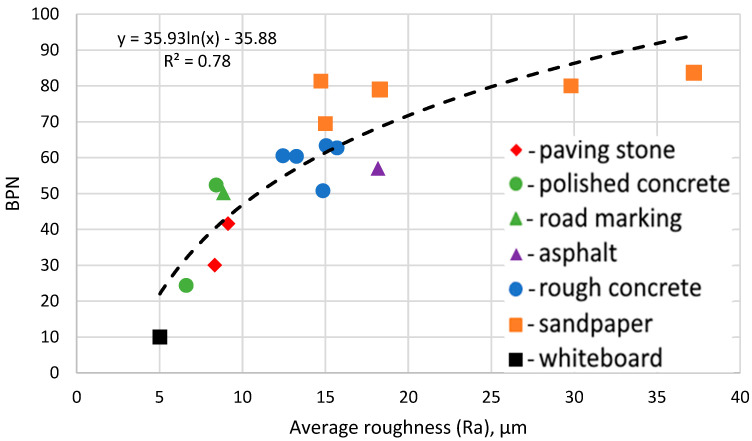
The correlation between BPN value and average roughness.

With the laser profilometer validated on place surfaces, 17 different surfaces with macrotexture were tested using the laser profilometer (Figure 5) and a British Pendulum Tester (Figure 6). The profilometer results were smothered and filtered according to the algorithm explained above. Each surface was tested 20 times, and average roughness based on the results was calculated. All results were compared, and the *S*-coefficient was optimised to maximise the R^2^ value. The final *S*-coefficient was 26. Test results after processing are shown in Figure 14.

During the roughness assessment, the standard deviation was calculated for the 20 replicate measurements of each surface type. The average standard deviation was 21.5% of the average result. A high standard deviation was obtained due to the limited horizontal range of the profilometer and high variations of the surface microtexture. However, a sufficient number of measurements can be taken to reduce any error during the assessment of any given surface, and this should be considered in future research.

A strong correlation between BPN and average roughness was found. This correlation did not depend on the type of material, and a difference in macrotexture did not affect it either. That validates the possibility of microtexture test application for the friction surveys. The R_a_ value, however, does not entirely describe the microtexture by ignoring the shape of asperities, which describes some difference between sandpaper and rough concrete on a graph. The difference, however, was not significant for practical measurements. The correlation obtained in this study (R^2^ = 0.78) was slightly lower than in other studies (Table 1). However, the surfaces were significantly more varied than for the other studies. That is, the data obtained in this study covers a broader range of surface roughness compared to all the studies detailed in Table 1, meaning a lower correlation between the profilometer results and the BPN was expected.

It can be seen that the optimal microtexture average roughness was generally in the range between 10 μm and 20 μm. Above 20 μm, microtexture does not significantly increase the BPN value, which reached a maximum value of 80 in the case of the sandpaper. Furthermore, values of microtexture below 10 μm resulted in a significant drop in the BPN value.

The obtained *S*-coefficient indicates that the wavelength threshold between microtexture and macrotexture, in terms of correlation between the British Pendulum Tester and the R_a_ value after macrotexture filtration, was approximately 0.3 mm. Those results correspond well to the PIARC classification, as shown in Figure 1 [2].

Taking into account the ready availability and economy of the proposed methodology, laser profilometry has the potential to be an effective tool for routine friction assessment when combined with a suitable macrotexture filtration algorithm. However, further testing on typical runway surfaces is required to assess the practical repeatability of the method and the influence of microtexture on the high-speed friction testing results obtained by continuous friction measurement equipment and other tools.

## 4. Conclusions

This study provides the required basis for the improvement in the friction measurement system by introducing microtexture measurements to runway surface friction assessment. This study focused on the design of a microtexture assessment algorithm that includes filtration and smothering techniques based on linear approximation and a laser profilometer. The laser profilometer model presented in this study was designed with economical and commonly available components and has a maximum vertical and horizontal resolution of 6 μm. The laser profilometer and microtexture assessment algorithm were validated and calibrated. This study revealed the following results:

- Laser profilometry testing equipment for the friction assessment can be economical and reliable.
- The optimal angle between the laser, camera, and surface was equal to 60°, which increases the vertical resolution of the profilometer without distorting the resulting profile.
- The proposed laser profilometry method results agreed with stylus-based roughness tester results, with a R^2^ coefficient of 0.99.
- A comparison of laser profilometer testing results to the British Pendulum Number of different pavement surfaces revealed that the average roughness had a good correlation with the British Pendulum Number (R^2^ = 0.78), which validates the friction assessment method based on texture testing.
- The filtration coefficient optimisation found that the wavelength threshold between microtexture and macrotexture, in terms of correlation between the British Pendulum Tester and average roughness, was approximately equal to 0.3 mm.
- Increasing microtexture roughness improves friction, but beyond 20 μm roughness has no significant effect on BPN value.

These findings show that cost-effective microtexture assessment can be used to assess friction. However, more research is needed to link texture measurements, including macrotexture measurements, to continuous friction measurement results. That research can be completed once the reliability and repeatability of the laser profilometer have been demonstrated on a range of real runway surfaces.

## Figures and Tables

**Table 1:** Correlation between friction and texture parameters in different studies.

№	Model	R^2^	Friction Assessment	Texture Assessment	Reference
1	$SN_{40}=18.6\cdot TXT+12.6$ $TXT=\frac{SF_{C}\times 1000}{CA}$ $sF_{c}=\frac{av\mathrm{e}rage\;peak\;height}{average\;peak\;width}$	0.70	Skid Number according to ASTM E 274-70	Microtexture shape factor and contact area based on macrotexture	[34]
2	Rubber friction theoretical model [35] with modified data from optical measurement	0.91 and 0.97	ViaTech and Wehner/Schulze machine	Optical measuring system data	[36,37]
3	$\begin{array}{c} Y_{Fr}=\beta_{0}+\beta_{macro}\cdot X_{macro}+\beta_{micro}\cdot X_{micro}\\ \;\;\;+\sum_{i=i}^{4}\beta_{i}X_{type\;i} \end{array}$	0.43–0.82	British Pendulum Number, Grip number, Dynamic friction test	Macrotexture and microtexture parameters, obtained by profilometry	[38]
4	$\mu =\varphi +\sum_{1}^{8}k_{i}\mu_{i}$	0.78	Grip Tester	Texture parameters, obtained by 3D scanning data	[32]
5	Rubber friction theoretical model [35] with modified data from 3D scanning	0.60	British Pendulum Number	3D scanning data	[39]
6	$S_{MI}=BPN\cdot 0.0102+0.846$	0.84	British Pendulum Number	Microtexture Index obtained by 3D scanning	[40]
7	$\begin{array}{cc} PTV_{predicted}= & -837.43+96.26\cdot S_{dq,MIC}-852.14\\ & \;\cdot S_{k,MIC}+10.41\cdot S_{mr2,MIC}+4.92\\ & \;\cdot S_{pc,MAC} \end{array}$	0.82	British Pendulum Number	Texture parameters, obtained by 3D scanning	[41]
8	Artificial neural network model	0.77–0.95	Dynamic Friction Tester	3D scanning data	[42]
9	Artificial neural network model	0.85	Sideway-Force Coefficient Routine Investigation Machine (SCRIM)	Sand patch test and 3D scanning	[43]
10	$BPN\left(AC\right)=9.235+1899.789\;R_{ami}+54.348\;\;R_{ama}$ $\begin{array}{cc} BPN\left(SMA\right)= & -91.573+10.938\;CR\\ & \;\;+6547.885\;R_{ami}+81.599\;\;R_{ama} \end{array}$ $\begin{array}{cc} BPN\left(BBTM\right)= & -194.201+4.787\;CR\\ & \;\;+2926.585\;R_{ami}+296.675\;\;R_{ama} \end{array}$	0.82–0.95	British Pendulum Number	Microtexture and macrotexture average roughness and rubber content for different mixes	[44]

**Table 2:** Sets of tests for profilometer verification.

Test Number	The Angle Between Camera and Surface (*α*), °	The Angle Between Laser and Surface (*β*), °	Theoretical Vertical Resolution of the Profilometer, μm	Coefficient of Determination Between the Roughness Tester and Profilometer (R^2^)
1	45	45	4.31	0.92
2	45	90	8.61	0.95
3	60	90	12.18	0.89
4	60	60	6.09	0.99

## Data Availability

The products presented in this article are available on request from the corresponding author.

## Nomenclature

*h*	µm	real height of the point on a profile
*h*′	µm	height of the point on a registered profile
*α*	°	angle between camera and surface
*β*	°	angle between laser and surface
*d_n_*	µm	distance between point n and macro-profile
*y_n_*	µm	*y*-coordinate of the point *n*
*x_n_*	µm	*x*-coordinate of the point *n*
*a_n_*	µm	*y*-intercept coefficient of an approximated line
*b_n_*	-	slope coefficient of an approximated line
*S*	points	filtration coefficient
*x_i_*	µm	*x*-coordinate of a point within the (*n* − *S*; *n* + *S*) or (*n* − *S*′; *n* + *S*′) range
*y_i_*	µm	*y*-coordinate of a point within the (*n* − *S*; *n* + *S*) or (*n* − *S*′; *n* + *S*′) range
*a*′*_n_*	µm	*y*-intercept coefficient of a line within the smothered profile
*b*′*_n_*	-	slope coefficient of a line within the smothered profile
*y*′*_n_*	µm	*y*-coordinate of a point *n* within the smothered profile
*S*′	points	smothering coefficient
*R_a_*	µm	average roughness of texture
*BPN*	-	British Pendulum Number
*R^2^*	-	coefficient of determination
